# Migraine-related disability and co-morbid depression among migraineurs in Ethiopia: a cross-sectional study

**DOI:** 10.1186/s12883-018-1095-3

**Published:** 2018-07-02

**Authors:** Biniyam Alemayehu Ayele, Yared Mamushet Yifru

**Affiliations:** Department of Neurology, College of Health Science, Addis Ababa Univeristy, PO BOX: 6396, Addis Ababa, Ethiopia

**Keywords:** Migraine, Depression, Disability, Co morbidity, PHQ-9, MIDAS

## Abstract

**Background:**

Migraine headache is a neurologic disorder which mainly affects younger and productive segment of population. Migraine not only causes pain; but also affects quality of life in terms of low productivity and economic loss. The main aim of this study was to examine migraine-related disability, co-morbid depression, and relationship between the two.

**Methods:**

A cross-sectional study was conducted among migraineurs who visited two neurology referral clinics. The study was conducted between June 1st 2016 to December 30^th^2016. Migraine disability assessment score [MIDAS] and patient health questionnaire [PHQ-9] were used to assess disability and depression, respectively.

**Results:**

A total of 70 patients participated in the study. Fifty-three (74.3%) of our study participants were women. Fifty one (72.9%) study participants were between age group 20–40 years. Migraine without aura was the most common subtype (70%); migraine with aura accounted for the other 28.6%. The mean (± SD) headache frequency and intensity was 23.4 ± 14.9 days and 7.4 ± 1.2 respectively. Major depressive disorder was common in this group (41.4%). The mean MIDAS and PHQ-9 scores were 46.7 ± 30 and 9.2 ± 4.4 respectively. More than two-thirds (74.3%) of our participants had severe disability. We found a statistically significant correlation between migraine-related disability and co morbid depression among our participants(*r* = 0.318, *p*-value = 0.007).

**Conclusion:**

The positive correlation observed between migraine-related disability and co-morbid depression warrant routine screening and treatment of disability and depression in migraineurs; In addition, the observed high degree of disability among our participants may indicate sub optimal treatment of these patients.

## Background

Migraine is a disabling neurologic disorder; characterized by recurrent and often unilateral headaches. Migraine causes substantial psychological and economic impact on the individual and society at large [1]. It is three times more common in women than men. Symptoms such as: nausea, vomiting, photophobia and phonophobia are often present in most migraineurs; and a few report osmophobia [2]. Migraine is common. According to the Global Burden of Disease survey (GBD 2010), its estimated prevalence was close to 15% among the general population [3]. In patients with migraine, recurrent episodes have the potential to progress to the more frequent and severe attacks of chronic migraine (CM), which affects 2.4% of the general, population [4]. Migraine headache is classified into two broad categories; migraine with aura and migraine without aura. Migraine without aura is a clinical syndrome characterized by headache and associated autonomic features but without any warning symptoms, also known as aura. Migraine with aura is characterized by focal neurological symptoms that may precede or sometimes accompany headache [5].

Ethiopia is the second most populous country in Africa, with a population of more than 100 million. Population-based studies done in Ethiopia in the past two decades have shown the prevalence of migraine headache to be between 3 to 17.7% [6, 7]. In a recent Ethiopian study of migraine in relation to psychiatric co-morbidities, participants with moderate to severe depressive symptoms had a 3-fold increased odds of migraine compared with those with minimal or no depressive symptoms, and the odds of migraine increased with increasing severity of depressive symptoms [8]. Migraine is documented as a major cause of disability worldwide. According to the World Health Report, migraine is the 19th leading cause of Years of Life with Disability. In addition, the global Burden of Headache reported migraine as the leading cause of disability among neurological disorders; and globally migraine was ranked as the seventh highest cause of disability [9–11].

It has long been known that migraine is associated with a number of medical and psychiatric conditions. One of the most common psychiatric co-morbidity is depression; which is reported in up to 80% of patients, especially in chronic migraine [12]. Patients with migraine are two to four times more likely to develop lifetime major depression as compared to those without migraine [13]. Different Studies suggest that the presence of depression in migraine patients significantly increase the level of disability that these patients experience in their daily life. As such, depression is associated with a greater incidence of severe disability. Patients with severe disability are six times more prone to have depression compared to those with lower disability [13, 14].

The Presence of clinical depression in a migraineurs is not only associated with more frequent and severe headaches, it also has the risk of transforming episodic migraine to chronic migraine. Chronic migraine is more disabling and often refractory to treatment [15]. The co-existence of depression can also adversely affect the quality of life by increasing the burden of the disease [15, 16]. We did this study with the main objective, to determine the relationship between migraine-related disability co-morbid depression among migraineurs having a follow-up at two neurology referral clinics in Addis Ababa, Ethiopia.

Even though multiple studies done elsewhere confirm the positive correlation between migraine-related disability and co-morbid depression, to the authors’ best knowledge, this is the first study of its kind to assess disability and depressive symptoms and their association among migraineurs in Ethiopia. As a result, we believe this study will be a baseline study for Ethiopia and other sub-Saharan countries.

## Methods

This is an observational, cross-sectional, hospital based study conducted between June 1st 2016 to December 30th 2016. Demographic variables included in this study were age, sex, marital status, educational status, occupation and religion. The study was conducted at Tikur Anbessa Specialized Hospital (TASH), a university teaching hospital and the only tertiary level referral hospital in Ethiopia; located at the heart of Addis Ababa and Zewditu Memorial Hospital (ZMH), a government referral hospital also located in Addis Ababa having affiliation to Addis Ababa University.

Data collection was started after obtaining formal approval letter from Institutional Review Board (IRB) of College of Health Sciences, Addis Ababa University. Inclusion criteria was all migraine patients attending both neurology referral clinics during the study period in which a diagnosis of migraine (both migraine with aura and migraine without aura) was made by a neurologist based on the International Classification of Headache Disorders (ICHD-3) and having age greater than 13 years (by tradition patients with age > 13 years are seen at adult outpatients clinic in our hospital) [5]. An exclusion criterion was migraine patients having additional diagnosis of other primary headache disorders like tension type headache (Tables 1 and 2).

**Table 1:** Diagnostic criteria for migraine without aura

A. At least five attacks fulfilling criteria B–D	
B. Headache attacks lasting 4–72 h (untreated or unsuccessfully treated)	
C. Headache has at least two of the following four characteristics: 1. Unilateral location 2. Pulsating quality 3. Moderate or severe pain intensity 4. Aggravation by or causing avoidance of routine physical activity (walking or climbing stairs)	
D. During headache at least one of the following 1. Nausea and/or vomiting 2. Photophobia and Phonophobia	
E. Not better accounted for by another ICHD-3 diagnosis.	

*Note. ICDH. The International Classification of Headache Disorders: 3rd edition. Cephalalgia. 2013; 33:9; 629–808*

**Table 2:** Diagnostic criteria for migraine aura:-

A. At least two attacks fulfilling criteria B–C	
B. One or more of the following fully reversible aura symptoms:	
1. visual	
2. sensory	
3. speech and/or language	
4. motor	
5. brainstem	
6. retinal	
C. At least two of the following four characteristics:	
1. At least one aura symptom spreads gradually over 5 min, and/or two or more symptoms occur in succession	
2. Each individual aura symptom lasts 5–60 min1	
3. At least one aura symptom is unilateral2	
4. The aura is accompanied, or followed within 60 min, by headache	
D. Not better accounted for by another ICHD-3 diagnosis, and transient ischemic attack has been excluded.	

*Note. ICDH. The International Classification of Headache Disorders: 3rd edition. Cephalalgia. 2013; 33:9; 629–808*

A total of 72 patients with confirmed diagnosis of migraine were interviewed. Two patients were excluded because of the co-occurrence of tension type headache with migraine headache. The demographic and clinical data were collected from structured survey questionnaire. Disability was assessed by Migraine Disability Assessment Score [MIDAS], well validated disability screening tool. MIDAS is simple to administer, easily interpreted and has been validated in population-based samples [17]. MIDAS is graded in to four grades; Grade I (little or no disability, scores range 0–5), grade II (mild disability, scores range 6–10), grade III (moderate disability, scores range 11–20), and grade IV (severe disability, 21 or greater).

The Patient Health Questionnaire [PHQ-9] was used to screen depression. PHQ-9 is a nine-question screening instrument for depressive symptoms based on Diagnostic and Statistical Manual of Mental Disorders (DSM) IV criteria to diagnose depression and also a validated tool in African population [18, 19]. PHQ-9 has also been validated in Ethiopia [20]. All the interviews were conducted by the two investigators; both neurologists. The statistical analysis was performed using SPSS version 20.0 computer program. Chi square test with *p* values and crude odds ratio (OR) with a 95% confidence interval (CI) were used to determine to statistical associations of selected variables.

A *p*-value < 0.05 was considered significant. Descriptive summaries were employed for socio-demographic and other clinical variables. Analytical statistics including bivariate analysis with Spearman’s correlation were performed to determine the correlation between; disability and depression.

## Results

Of the seventy migraine patients interviewed at the two Neurology clinics in Addis Ababa, three-fourths (74.3%) were female. More than two third (72.9%) of our study participants were between age group 20–40 years, 5.7% were < 20 years and 18.6% of them were > 40 years (Table 3). Thirty-three (47.1%) were married and 8 (11.4%) of them were divorced. Thirty four (48.6%) patients were government employees, 9 (12.9%) were students and only 2(2.9%) were unemployed. One-third (28.6%) of the study participants had a secondary school education and 27.1% had a Bachelor’s degree or above educational level (Table 3).

**Table 3:** Frequency distribution of socio-demographic characteristics of migraine patients. *N* = 70

Variables	Number	Percent
Gender		
Male	18	25.7
Female	52	74.3
Age groups		
14–20	4	5.7
20–30	28	40
30–40	25	35.7
40–65	13	18.6
Marital status		
Single	23	32.9
Married	33	47.1
In a relationship	5	7.1
Divorced	8	11.4
Widowed	1	1.5
Occupational status		
Government employee	34	48.6
Student	9	12.9
Private business	13	18.6
House wife	7	10.0
Farmer	2	2.9
Jobless	2	2.9
Other	3	4.3
Educational status		
Illiterate	2	2.9
Read and write only	3	4.3
Primary education (1–8)	8	11.4
Secondary education (8–12)	20	28.6
Diploma	18	25.7
Degree and above	19	27.1

Among women migraineurs, 31.5% experienced worsening or occurrence of their migraine headache during menses. The majority of our study participants (70%) had migraine without aura, while 28.6% of our participants reported auras of different type prior to their migraine attack. Whether patients with aura did have at times headache episodes without accompanying aura was not specifically queried. One patient found to have diagnosis of ophthalmoplegic migraine (1.4%).

The majority of participants had a diagnosis of migraine by a physician less than five years ago (54.3%), while24.3% had it for 5–10 years. The rest of our participants (21.4%) had migraine for more than 10 years (Table 4). Among our study participants 31.5% of them are on medications for migraine prophylaxis; including 25.1% on Amitriptyline, 5.9% on propranolol and 0.5% on Imipramine (Table 4).

**Table 4:** Frequency distribution of headache-related characteristics of migraine patients. *N* = 70

Migraine headache worsening/occurring during Menses		%
	Yes	31.5
	No	68.5
	Total	100
Subtype of Migraine	Migraine without aura	70.0
	Migraine with aura	28.6
	Ophtalmopelgic migraine	1.4
	Total	100
Duration of Migraine headache	< 05 years	54.3
	5–10 years	24.3
	> 10 years	21.4
	Total	100
Migraine prophylaxis	Amitriptyline	25.1
	Propranolol	5.9
	Imipramine	0.5
	Total	31.5

The mean PHQ-9 score in our study was 9.2 ± 4.4. Severe depressive symptoms were present in 14.3% of study participants, while 45.7% had a PHQ-9 score between 5 and 9, indicative of mild depressive disorder. Many (41.4%) of our participants fulfilled criteria for major depressive disorder (Table 5). The mean MIDAS score was 46.7 ± 30. Over two-third of our patients (74.3%) had severe disability, defined as MIDAS score ≥ 21, while 15.7%, had moderate and 8.6% mild disability. Only 1.4% of our study participants had little or no disability (Table 6).

**Table 5:** Patient Health Questioners (PHQ-9) score of migraine patients. *N* = 70

PHQ-9	Mean(SD)	9.2 ± 4.4
Minimal (0–4)	%	12.9
Mild (5–9)	%	45.7
Moderate(10–14)	%	27.1
Severe(> = 15)	%	14.3

**Table 6:** Migraine Disability Assessment Scale (MIDAS) score of migraine patients. *N* = 70

MIDAS Scoring	Mean(SD)	46.7 ± 30
Little or no disability (0–5)	%	1.4
Mild disability (6–10)	%	8.6
Moderate disability (11–20)	%	15.7
Severe disability (> = 21)	%	74.3

Spearman’s correlation was used to examine associations between co-morbid depression and disability. There was a significant (*p* = 0.007) correlation between severe disability (Grade IV) and depression (PHQ-9, Grade II-IV) for both minor and major depressive disorders, compared to non-depressed patients (PHQ-9, Grade I). Spearman’s coefficient of this correlation is *r* = 0.318, indicating positive correlation between depression and severe disability (Table 7). Depression was associated with occurrence of severe disability; severe disability was found to be 8 times more common in patients with depression in comparison to those having no depression. Depressed migraine patients were five times more likely to have severe disability (*p* = 0.007) compared to non-depressed patients. In our study; we also found a statistically significant (*p* = 0.002) association between migraine-related disability and migraine occuring during menses (Table 8).

**Table 7:** Correlation between degree of disability and depression among migraine patients. *N* = 70

	MIDAS score		*P*-value	Spearman coefficient
	MIDAS Grade < IV	MIDAS Grade IV		
PHQ-9 Grade II-IV	47	14	0.007	0.318
PHQ-9 Grade I	5	4		
Total	52	18		70

Spearman coefficient = 0.318; indicative of positive correlation. Statistically significant *P*-value < 0.001, 95% CI

**Table 8:** Association between disability and migraine during menses among migraine patients. *N* = 70

	MIDAS score		Total	*P*-value
	MIDAS Grade < IV	MIDAS Grade IV		0.02
Headache During menses				
Yes	7	12	19	
No	34	17	51	
Total	41	29	70	

Statistically Significant *P*-value < 0.05, 95% CI

## Discussion

Most of our participants were females, and two-thirds of them had migraine without aura. Significant proportion of the study participants fulfilled the criteria for major depressive disorders, and many of them had severe disability scores. We also found a strong correlation between disability and co- morbid depression among migraineurs. Most of the migraine- related clinical characteristics, like female preponderance, high proportion of migraine without aura observed in our study, are comparable to prior published studies on migraine from both developing and developed countries [21].

We observed one patient with diagnosis of ophthalmoplegic migraine in our study; which we also saw in other reported cases from Ethiopia in the past few years [22, 23]. When we compare our findings with the study done by Pavlović 2015 and his colleagues, in which 60% of women with migraine reported an association between migraine attack and menses [24], our finding show much lower than this, only one third of our participants reported an association between migraine attack and menses; which could be attributable to lack of awareness among our patients about the possible association between menses and migraine and not using the headache diary, which often is helpful in tracking such precipitating causes.

Close to half of our participants are government employees (Table 3) and their diagnosis of migraine was made in less than five years (Table 4). This might be due to the fact that the study was done in the capital city, Addis Ababa, where better neurologic service was started only recently.

Two factors may explain the higher prevalence of migraine-related disability among our study participants. First, the fact that this is a hospital based study and most likely many of our patients had prolonged treatment by general practitioners in other facilities then referred for better assessment and treatment by a neurologist. Secondly, the suboptimal treatment and lack of screening for management of disability and co -morbid events like depression may also contribute to the higher level of morbidity.

An interesting finding in this study is high proportion of our study participants were on amitriptyline for migraine prophylaxis, yet significant proportion had persistent major depressive disorder (MDD). This may be due to use of low doses of amitriptyline, which is inadequate to treat depression as well as lack of psychotherapy.

Regarding co-morbid depression based on PHQ-9 score; the mean PHQ-9 (Table 5) of our study is comparable to the finding from Canadian study, in which the mean PHQ-9 score was 8 [25]. Among our study participants, significant proportion of them fulfilled the criteria for Major depressive disorder with PHQ-9 ≥ 10 (Table 5), which is comparable to one meta-analysis which reported the incidence of depression in migraineurs to be between 8.6 to 47.9% [26].

Our mean MIDAS score (Table 6) with higher than those reported from studies done in Turkey (19.3 ± 12.3) and Italy (12 ± 8.2) [27, 28]. More than two third of our study participants had severe disability with a MIDAS score ≥ 21 (Table 6), which is much higher than the result from a similar study from Turkey, in which severe disability was noted in 40%. One study done in South Korea reported a much higher MIDAS score of 54.1 ± 49.9, mainly among chronic migraine patients in their study [29].

In our study, depression was associated with greater incidence of severe disability. Our finding is comparable to those results reported from South Korea [29] and another study reported by JL Brandes et al. [30] which showed patients with MDD had significant association with severe disability [MIDAS Grade IV], compared to non-depressed migraineurs. We also found a statistically significant (p 0.02) association between migraine-related disabilities in migraine occurring during menses; this finding highlights the need to routinely look for aggravation of migraine during menses, so that we may be able to optimize both abortive and preventive migraine treatment in such situation.

Limitations of our study include a small sample size and the fact that this is hospital-based study with likelihood of over representation of more severe cases. As a result we acknowledge the limited generalizability of these findings to the whole population. In addition, for both the screening tools we were dependent on patient’s ability to remember symptoms in the past two weeks for depression screening and three months for disability, which could have introduced recall bias.

## Conclusion

We conclude from our analysis that there is a higher proportion of severe disability and co-morbid depression with positive correlation between the two in migraine. Our findings show the importance of screening for disability and depression in migraine patients, as this might guide our management approach and impact their overall therapeutic outcome.

Finally, this study points to the need for large scale longitudinal, observational study in Ethiopia to evaluate the relationship between migraine-related disability and co-morbid depression.

## Abbreviations

MIDAS: Migraine Disability Assessment Score
PHQ-9: Patient Health Questioner 9
TASH: Tikur Anbessa Specialized Hospital
ZMH: Zewditu Memorial Hospital
MDD: Major Depressive Disorder
